# The Ventilatory and Diffusion Dysfunctions in Obese Patients with and without Obstructive Sleep Apnea-Hypopnea Syndrome

**DOI:** 10.1155/2020/8075482

**Published:** 2020-02-10

**Authors:** Sonia Rouatbi, Ines Ghannouchi, Rim Kammoun, Helmi Ben Saad

**Affiliations:** ^1^Laboratory of Physiology and Explorations, Faculty of Medicine Sousse, University of Sousse, Sousse, Tunisia; ^2^Heart Failure (LR12SP09) Research Laboratory, Farhat Hached Hospital, Sousse, Tunisia

## Abstract

**Objective:**

To analyze the ventilatory and alveolar-capillary diffusion dysfunctions in case of obesity with or without an OSAS.

**Methods:**

It is a cross-sectional study of 48 obese adults (23 OSAS and 25 controls). Anthropometric data (height, weight, and body mass index (BMI)) were collected. All adults responded to a medical questionnaire and underwent polysomnography or sleep polygraphy for apnea-hypopnea index (AHI) and percentage of desaturation measurements. The following lung function data were collected: pulmonary flows and volumes, lung transfer factor for carbon monoxide (DLCO), and fraction of exhaled nitric oxide (F_e_NO).

**Results:**

Obesity was confirmed for the two groups with a total sample mean value of BMI = 35.06 ± 4.68 kg/m^2^. A significant decrease in lung function was noted in patients with OSAS compared with controls. Indeed, when compared with the control group, the OSAS one had a severe restrictive ventilatory defect (total lung capacity: 93 ± 14 vs. 79 ± 12%), an abnormal DLCO (112 ± 20 vs. 93 ± 22%), and higher bronchial inflammation (18.40 ± 9.20 vs. 31.30 ± 13.60 ppb) (*p* < 0.05).

**Conclusion:**

Obesity when associated with OSAS increases the severity of pulmonary function and alveolar-capillary diffusion alteration. This can be explained in part by the alveolar inflammation.

## 1. Introduction

Obesity has reached epidemic proportions worldwide (12% of the world's population) [1]. Obesity, vascular dysfunction, and obstructive sleep apnea syndrome (OSAS) are associated disorders. The role of adipo(cyto)kines in systemic inflammation and vascular dysfunction has been proved by many studies [2]. Obesity is a risk factor of OSAS, defined as an apnea-hypopnea index (AHI) >10/h [3–6]. OSAS currently represents a real public health problem, with an adult prevalence of 2–4% [4, 6, 7]. Obstructive apnea corresponds to a stop of the naso-oral ventilation with persistence of thoraco-abdominal movements [3–8]. Pharyngeal hypercollapsibility, often seen in case of obesity, is one of the many causes of OSAS [9]. Polysomnography in the sleep laboratory remains the main tool for diagnosis of OSAS [9–11]. The OSAS can have many serious consequences: metabolic, behavioral, or cardiovascular (coronary insufficiency and hypertension) [8, 9, 12–15]. These latter consequences are common in patients with OSAS, but the underlying mechanisms of this association are unknown. Several hypotheses evoke an alteration of endothelial tissue as a mechanism of these vascular complications in case of OSAS [16]. Thus, the objective of this work is to analyze the ventilatory mechanism and alveolar-capillary diffusion dysfunctions in case of obesity with or without an OSAS.

## 2. Population and Methods

### 2.1. Study Design

This was a cross-sectional study conducted in the physiology and functional exploration laboratory. The studied sample was composed of 48 obese adults divided into two groups: an obese control group (G_1_, *n* = 25) free from any respiratory disease and an obese OSAS group (G_2_, *n* = 23). OSAS patients had the following characteristics: age between 20 and 65 years, obesity, and a confirmed OSAS by polysomnography with an AHI ≥ 10. Subjects with one or more of the following criteria were excluded from the study: respiratory infection of the upper or lower respiratory tract, asthmatic disease or chronic obstructive pulmonary disease, known neuromuscular pathology, upper airway abnormality, imperfect performance of required breathing maneuvers, and smoking >10 pack-year [17].

### 2.2. Survey

All subjects responded to a standardized questionnaire seeking inclusion and noninclusion criteria, respiratory function signs (cough, dyspnea, expectoration, snoring, and daytime sleepiness), and anthropometric data (sex, age (years), weight (kg), height (m), and body mass index (BMI, kg/m^2^)). Based on BMI values, three classes of obesity were defined [18, 19]: class 1 (BMI between 30 and 34.9 kg/m^2^), class 2 (BMI between 35 and 39.9 kg/m^2^), and class 3 (BMI greater than 40 kg/m^2^).

### 2.3. Functional Respiratory Explorations: Total Body Plethysmography

Total body plethysmography was performed for all participants in the study using a plethysmograph (ZAN 500, Messgeraete GmbH2000, Germany). Ventilatory data were interpreted according to the international recommendations [20]. The total body plethysmography allows the realization of a flow-volume curve and the measurement of ventilatory flows and pulmonary volumes. The measured data were the following: forced expiratory volume at the first second (FEV_1_, l), slow vital capacity (SVC, l), forced vital capacity (FVC, l), FEV_1_/VC ratio (%), maximal mid-expiratory flow (MMEF, l/s), forced expiratory flow at *x*% of FVC (FEF_25%_ and FEF_50%_, l/s), total lung capacity (TLC, l), and residual volume (RV, l). These data were considered diminished when they were below the lower limit of normal (LLN). The LLNs were determined from the specific reference values of the Tunisian population. Different ventilatory patterns were defined: (i) a proximal obstructive ventilatory defect is defined when the FEV_1_/VC or FEV_1_/FVC ratios were lower than the LLN [20]; (ii) a distal obstructive ventilatory defect is defined when the FEV_1_/FVC ratio is normal, the FVC is normal, and FEF_25%_ or FEF_50%_ or MMEF were less than the LLN [20]; (iii) a TLC lower than the LLN defines a restrictive ventilatory defect [20].

### 2.4. Carbon Monoxide Transfer Capacity (DLCO)

DLCO (mmol/KPa/min) was measured by the inspiratory apnea method. These data are considered diminished when they are lower than the LLN [20].

### 2.5. Polysomnography

Respiratory events are apneas and hypopneas. Obstructive apnea is defined as naso-oral airflow arrest for at least 10 seconds with persistent ventilatory efforts during apnea [3, 5, 8]. Hypopneas are defined as a reduction of more than 50% of the oro-nasal flow amplitude during 10 seconds, accompanied by 3% desaturation and/or arousal. The AHI is the number of apneas and hypopneas per hour of sleep [21, 22]. The severity of OSAS is defined according to the value of AHI [3, 5]: light (AHI < 15), moderate (15 < AHI < 30), and severe (AHI > 30) [23]. Polysomnographic scoring and staging were based on Rechtschaffen and Kales study, and episodes of arousals were assessed according to the guidelines in the previous studies [24].

### 2.6. Measurement of Exhaled Fraction of Nitric Oxide (F_e_NO)

F_e_NO was measured by the Medisoft HypAir method using an electrochemical analyzer (Medisoft, Sorinnes, Belgium). It was based on the chemiluminescence method [25]. The instrument was calibrated and used according to the manufacturer's instructions. The measurement of F_e_NO was made following the international recommendations [25]. Three acceptable measurements were taken at a flow rate of 50 ml/s at 15 minutes as recommended [25]. The average of the three values was used. F_e_NO was expressed in parts per billion (ppb), which is the equivalent of nanoliter per liter [25].

### 2.7. Statistical Analysis

The statistical analysis was performed using the Statistica software (Statistica Kamel version 6.0, Stat Soft, France). In a first step and after checking the normal distribution of the studied data, the means (standard deviations) of all the quantitative data (anthropometric and ventilatory) for both groups were determined. The Mann–Whitney *U* test was used to compare the quantitative data (respiratory data) of the two groups. Comparison of categorical data (sex-ratio, smoking habits, hypertension, diabetes, and so on) between the two groups was set by the chi-squared test. The degree of significance was set at “*p*” lower than 0.05.

## 3. Results

Forty-eight participants were included in the study. They were divided into two groups: *G*_1_ (25 obese controls, 16 males) and *G*_2_ (23 obese OSAS, 16 males). The OSAS patients had an Epworth sleepiness score of 13.78 ± 4.92, an AHI > 10 with an oxygen saturation average of 89 ± 6%, and a number of desaturations per night of sleep at 443 ± 148. The G_1_ group had an AHI < 10.

The anthropometric data of the two groups are shown in Table 1. Twenty-one OSAS patients and the entire G_1_ group had obesity, and two OSAS patients were overweight. The two groups were matched for weight, height, sex, and BMI.

Twenty-tow participants (12 from the OSAS group) were active smokers. The comparison of smoking habits between the two groups showed no significant difference. Fourteen participants (10 from the OSAS group) had an arterial hypertension. Twenty participants (10 the OSAS group) had diabetes mellitus.  Table 2 summarizes the respiratory functional data of the two groups.

A significant decrease in lung function was noted in patients with OSAS compared with controls. Proximal (FEV_1_ expressed in liters and in percentage) and distal (MMEF, FEF_25%_, and FEF_50%_) flows' values were significantly lower in OSAS compared with controls. Five OSAS patients and no participant from the control group had a proximal obstructive ventilatory defect. The OSAS group had a tendency to a pulmonary restriction (low pulmonary volumes: SVC, FVC, and TLC). A restrictive ventilatory defect was present in 26 participants (16 from the OSAS group). An abnormal DLCO was found in 10 participants (8 from the OSAS group). The degree of bronchial inflammation (judged by F_e_NO) was significantly greater in the OSAS group than in the control one and was correlated with the OSAS degree of severity.

## 4. Discussion

This study showed that obesity when associated to OSAS increased the risk of altered pulmonary function with a decrease in DLCO. This result can be explained by both alveolar inflammation (increased F_e_NO) and vascular dysfunction.

The group of nonapneic obese was selected from a group of participants who were suspected having OSAS and whose polysomnography or polygraphy did not confirm this diagnosis. This group was used to determine the effect of OSAS alone on respiratory and cardiovascular functions by comparing OSAS obese patients with nonOSAS obese participants.

The OSAS group was selected after the confirmation of an OSAS by polysomnography. All respiratory and vascular functional explorations were performed by the same operator and at the same timing, in the morning for all participants, to respect the reproducibility of the measurements and to avoid circadian variations in respiratory function.

The OSAS group (*n* = 23) had a male predominance which is often found in case of OSAS [10, 26–28]. Young et al. [29] estimated that 93% of apneic females were undiagnosed. In addition, male predominance may be related to anatomical factors at upper airways: the increase in neck circumference and the important collapsibility of upper airways in males [30–32]. After menopause, this difference tended to disappear because of the disappearance of the protective hormonal climate of the female [32]. It has been reported that testosterone increased the collapse of upper airways and that progesterone played a protective role in maintaining good upper airway permeability [33].

The average age of the OSAS patients was 50 ± 9 years. In fact, the majority of patients with OSAS were older than 50 years [7, 34]. These results confirmed the accepted classical notion that the prevalence of OSAS increased with age [7, 33, 35, 36]. Durán et al. [5] showed that the prevalence of OSAS increased with age regardless sex with an odds ratio of 2.2 every 10 years. Age-related anatomical and histopathological changes in the pharynx led to the increased collapses (loss of elastic tissue) of the upper airways, which may explain the increased prevalence of OSAS with age [7, 32, 36]. Indeed, this hypercollapsibility associated to a decrease in muscle tone at upper airways during sleep was responsible for pharyngeal wall vibration and OSAS [36]. Planchard et al. [7] explained the sleep-related respiratory disturbances in apneic elderly patients to the aging of the ventilatory control and the thoracic mechanical performance.

In this study, all OSAS patients were obese with an average BMI of 35.78 ± 4.72 kg/m^2^. Obesity, especially in its massive or android form, is a major risk factor for OSAS [13, 14]. Indeed, a 10% of gain in body weight could predict an increase in AHI of 32%. This modification can be explained by the anatomical modifications of upper airways. Obesity is responsible of an increase in the compliance of the pharyngeal walls and the presence of external compression of the pharynx by the peripharyngeal fatty deposits [13, 14]. Abdominal fat found in android obesity could also play an important role in OSAS [4]. Indeed, since the functional residual capacity (FRC) is reduced in obese patients, contraction of the diaphragm can cause significant intrathoracic depression at the beginning of inspiration, which can lead to pharyngeal collapse [30, 36].

Spirometric data showed an obstructive ventilatory defect in 12 obese OSAS patients and 10 obese controls. The comparison between OSAS and control groups showed a significant lower FEV_1_ (*L* and %) in the OSAS group. This could be explained by the rise in oxidative stress during OSAS leading to a decrease in NO synthesis by pulmonary tissue and causing bronchial muscles relaxation defect [34, 37, 38]. However, FEV_1_ was considered by several authors to be an unsuitable tool for assessing the functional impact of OSAS since these data did not show a significant difference between participants with and without OSAS during their studies [11, 28, 39]. MMEF, FEF_25%_, and FEF_50%_ are the data that provide information on small airway obstruction. However, these data depended on the expiratory effort and especially the participants' cooperation, which was often difficult to obtain [20]. In the present study, MMEF, FEF_25%_, and FEF_50%_ were lower in the obese OSAS patients than in the obese controls. This can be explained by obesity that reduced lung volumes by pulmonary restriction and so decreased distal flow rates [21]. The restrictive ventilatory defect was objectified in 10 controls and 16 OSAS. Morbid obesity is associated with a decrease in static and dynamic lung volumes and an alteration of gas exchange and ventilatory mechanics. The most severe obese patients had a restrictive involvement characterized by a decrease in SVC, FRC, TLC, and RV [38, 39]. In the present study, SVC, FVC, and TLC were significantly lower in the OSAS group than the control one. DLCO was significantly lower in the OSAS group when compared with the control group. This result can be explained in part by bronchial inflammation and endothelial dysfunction. Different from our results, Hoffstein and Oliver [40] found a higher DLCO in 1296 apneic patients. Doré and Orvoën-Frija [19] concluded that apneic or obese nonapneic patients had an increased DLCO. In this study, the absence of DLCO elevation could be attributed to the association of two opposite mechanisms occurring during OSAS: (i) an increase in pulmonary capillary blood volume due to obesity and an increase in cardiac output linked to the hyperactivity of the sympathetic system, this latter tends to increase the DLCO; (ii) an alteration of the alveolar-capillary membrane which tends to reduce the DLCO. Indeed, during the course of OSAS in obese patients, an increase in the atherosclerosis and the inflammatory manifestations causing an alteration of the pulmonary exchanger was often noted [14, 15, 41]. The degree of bronchial inflammation was significantly greater in the OSAS group than the control group. The F_e_NO value correlated with the severity of OSAS. This increase in F_e_NO in obese OSAS could be caused by repetitive apnea and hypoxemia during sleep [37].

In the present study, arterial hypertension is significantly more frequent in the OSAS group than the obese control group. Many studies provide direct evidence of the bioavailability of NO that is reduced in OSAS patients with or without cardiovascular diseases. OSAS negatively affects endothelial regulation of peripheral vasomotricity [12, 16, 37, 42]. Hypoxemia resulting from repeated apneas does not have the same effect on bronchial tissue and vascular endothelium. At the bronchial tree, it was responsible of an increase in NO following inflammation of the bronchial wall (the origin is the bronchial epithelium). At the vascular level, this hypoxemia reduced the production of NO by vascular smooth muscle [37, 43]. Several hypotheses were advanced to explain hypertension in apneics: sleep fragmentation, intermittent hypoxemia, and sympathetic activation were the most validated. Yannoutsos et al. [44] objectified the responsibility of endothelial dysfunction in the occurrence of hypertension. It is well known that OSAS is associated with notable nonrespiratory morbidity, including an elevated prevalence of metabolic syndrome, arterial hypertension, insulin resistance, type 2 diabetes, and cardiovascular illnesses, such as transient ischemic attacks, stroke, cardiac arrhythmias, myocardial infarction, and pulmonary hypertension [45]. Insulin secretion increases the endogenous release of the potent vasodilator NO from the endothelium [45]. Circulating exosomes facilitate important intercellular signals that modify endothelial phenotype and thus emerge as potential fundamental contributors in the context of OSAS-related endothelial dysfunction [46]. Exosomes may not only provide candidate biomarkers but are also a likely and plausible mechanism toward OSAS-induced cardiovascular disease [46]. Recently, it was shown that levels of 8-isoprostane, though not exhaled NO, distinguish children with OSAS from those with primary snoring or healthy, correlate with disease severity. and closely predict OSAS in the whole sample observed [47].

This study presents some limitations: first, the sample size which was reduced to 48 due to the poor cooperation of participants in performing the respiratory maneuvers; the sample size of this study appeared to be satisfactory compared with that noted in the literature [26, 27].

## 5. Conclusion

It is confirmed that obesity and OSAS are, when associated, a major risk factor to decreased ventilation and diffusion lung functions with a trend to bronchial inflammation.

## Figures and Tables

**Table 1:** Anthropometric and clinical characteristics of the two obese groups.

	Control group (*n* = 25)	OSAS group (*n* = 23)	Total sample (*n* = 48)	*p*
Male (number)	16	16	32	0.682 (ns)
Age (years)	**43.53** **±** **9.60**	**50.08** **±** **9.28**	**46.61** **±** **9.92**	**0.019 ** ^*∗*^
Weight (kg)	97.00 ± 12.93	100.00 ± 13.20	98.40 ± 13.01	0.264 (ns)
Height (m)	1.68 ± 0.09	1.67 ± 0.09	1.67 ± 0.09	0.909 (ns)
Body mass index (kg/m^2^)	34.42 ± 4.63	35.78 ± 4.72	35.06 ± 4.68	0.179 (ns)
Smoking habits (yes/no)	10/15	12/11	22/26	0.397 (ns)
Diabetes mellitus (yes/no)	10/15	10/13	20/28	0.807 (ns)
Arterial hypertension (yes/no)	**4/21**	**10/15**	**14/34**	**0.036 ** ^ѳ^

ns, not significant difference; OSAS, obstructive sleep apnea syndrome. ^*∗*^*p* value < 0.05: comparison between controls and OSAS groups by the Mann–Whitney *U* test. ^ѳ^*p* value < 0.05: comparison between controls and OSAS groups by the chi-squared test.

**Table 2:** Respiratory functional data of the two obese groups.

	Control group (*n* = 25)	OSAS group (*n* = 23)	Total sample (*n* = 48)	*p*
FEV_1_ (L)	3.26 ± 0.70	2.59 ± 0.75	2.95 ± 0.79	0.005
FEV_1_ (%)	99 ± 12	83 ± 15	91 ± 16	<0.001
FEF_50%_ (L/s)	4.45 ± 1.14	3.72 ± 1.21	4.11 ± 1.21	0.057
FEF_50%_ (%)	98 ± 23	85 ± 26	92 ± 25	0.057
FEF_25%_ (L/s)	1.43 ± 0.50	1.20 ± 0.57	1.33 ± 0.54	0.217
FEF_25%_ (%)	74 ± 22	69 ± 35	72 ± 29	0.412
MMEF (L/s)	3.51 ± 0.90	2.99 ± 0.93	3.26 ± 0.94	0.062
MMEF (%)	89 ± 19	79 ± 26	84 ± 23	0.138
SVC (L)	3.98 ± 0.90	3.25 ± 0.96	3.64 ± 0.99	0.017
SVC (%)	98 ± 14	83 ± 12	91 ± 16	<0.001
FVC (L)	4.00 ± 0.94	3.14 ± 1.02	3.60 ± 1.06	0.006
FVC (%)	100 ± 13	83 ± 14	92 ± 16	<0.001
FEV_1_/FVC (%)	82 ± 6.	79 ± 9	81 ± 8	0.412
RV (L)	1.66 ± 0.50	1.65 ± 0.72	1.66 ± 0.61	0.525
RV (%)	90 ± 22	84 ± 32	87 ± 27	0.241
TLC (L)	**5.65** **±** **1.21**	**4.76** **±** **1.28**	**5.23** **±** **1.31**	0.018
TLC (%)	**93** **±** **14**	**79** **±** **12**	**87** **±** **15**	<0.0001
DLCO (mmol/KPa/min)	**10.70** **±** **2.40**	**8.70** **±** **2.40**	**9.80** **±** **2.60**	0.008
DLCO (%)	**112** **±** **20**	**93** **±** **22**	**103** **±** **23**	0.001
F_e_NO (ppb)	**18.40** **±** **9.20**	**31.30** **±** **13.60**	**24.85** **±** **11.40**	<0.0001

DLCO, carbon monoxide transfer capacity; FEF_*x*%_, forced expiratory flow at *x*% of FVC; F_e_NO, fraction of exhaled nitric oxide; FEV_1_, forced expiratory volume at the first second; FVC, forced vital capacity; MMEF, maximal mid-expiratory flow; ns, not significant difference; RV, residual volume; SVC, slow vital capacity; TLC, total lung capacity; %, percentage of predicted value; *p*, comparison between controls and OSAS groups by the Mann–Whitney *U* test.

## Data Availability

The data used to support the findings of this study are available from the corresponding author upon request.
